# The Antioxidant Capacity and Anti-diabetic Effect of *Boswellia serrata* Triana and Planch Aqueous Extract in Fertile Female Diabetic Rats and the Possible Effects on Reproduction and Histological Changes in the Liver and Kidneys

**Published:** 2012-10-07

**Authors:** Mohamad Ebrahim Azemi, Foroogh Namjoyan, Mohammad Javad Khodayar, Forouzan Ahmadpour, Azam Darvish Padok, Marziyeh Panahi

**Affiliations:** 1 Medicinal Plant Research Center, Department of Pharmacognosy, School of pharmacy, Ahvaz Jundishapur University of Medical Sciences, Ahvaz, IR Iran; 2 Marine Pharmaceutical Research Center, Pharmacognosy Department, School of pharmacy, Ahvaz Jundishapur University of Medical Sciences, Ahvaz, IR Iran; 3 Toxicology Research Center, Department of Toxicology-Pharmacology, School of Pharmacy, Ahvaz Jundishapur University of Medical Sciences, Ahvaz, IR Iran; 4 School of Pharmacy, Ahvaz Jundishapur University of Medical Sciences, Ahvaz, IR Iran; 5 Department of Anatomy, School of Medicine, Ahvaz Jundishapur University of Medical Sciences, Ahvaz, IR Iran

**Keywords:** *Boswellia*, Hypoglycemic Agents, Diabetes Complications, Rats, Antioxidants, Histology

## Abstract

**Background:**

*Boswellia serrata* has been used in a wide variety of diseases, including diabetes mellitus and inflammatory diseases.

**Objectives:**

This study focused on the effects of *Boswellia serrata* aqueous extract on blood glucose and the complications of diabetes in the liver and kidneys and examined the impact of plant on reproduction in diabetic rats.

**Materials and Methods:**

The antioxidant capacity of plant extract was performed using FRAP assay. Diabetic and control rats were administered 200, 400, and 600 mg/kg *Boswellia serrata* extract. Vaginal plaque was mentioned as a positive sign of pregnancy ;and treatment started with extract or vehicle from 1th to 17th day of gestation by gastric gavage. Blood glucose was measured during 17 days.

**Results:**

The Administration of *Boswellia serrata* in diabetic rats significantly decreased the level of blood glucose and HbA1c after 17th days (*P* ≤ 0.01). In diabetic group that received no treatment, the abortion of fetus spontaneous was 19.14%. The percentage of absorptions significantly was elevated in vehicle-treated diabetic rats, in comparison with vehicle- treated healthy rats. In the diabetic group, separated necrosis of hepatocytes, anarchism of liver plates, and lymphocytic inflammation were improved. Diabetic complications were not seen and the severity of damage was reduced. These damages include: lymphocytic inflammation in the port areas, irregularities, apoptosis of liver cells, and dilatation of the sinusoids.

**Conclusions:**

The results suggest that *Boswellia serrata* extract has the antidiabetic effects and can prevent the complications of diabetes in the kidneys and liver.

## 1. Background

Diabetes is a metabolic disorder resulting from insulin resistance or occurring impaired insulin secretion, therefore a diabetic patient will suffer from chronic hyperglycemia (1). Chronic high blood glucose in diabetes leads in advanced glycation end-products which will subsequently produce reactive oxygen species. The free radicals originating from damaged cell membranes and lipid peroxidation, causes irreversible damage to the liver, kidneys, eyes, nervous system, cardiovascular and other parts of the body (2). The liver is the organ that is severely damaged in diabetes.3 These damages and disorders include inflammation, necrosis, fibrosis of non-alcoholic fatty liver disease, cirrhosis, hepatocellular carcinoma, hepatitis, and acute liver failure (3). Impaired glucose and protein metabolism in diabetes can also lead to dysfunction and structure damages in kidney called diabetic nephropathy which causes the glomerular basement membrane thickening and increases the kidney interstitial tissue (4).

Diabetes is considered as an important reason for increasing malformations ratio (5) of fetus to 10 times and the risk of fetus death to 5 times (6). Malformations as a result of maternal diabetes occur at high rate including: spinal bifida, structural brain anomalies, anencephaly, hydrocephalus, neuropathic bowel and bladder, sexual dysfunction, skeletal deformation, endocrine disorder and other central nervous system defects (7). Good control of diabetes can reduce complications during pregnancy. The use of medicinal plants to treat a wide variety of diseases, including inflammatory diseases, diabetes mellitus, and many disorders in the liver and the kidneys are growing (8).

*Boswellia serrata* Triana & Planch (B.S) is a moderate-to-large branching tree, were found in India, North Africa and the Middle East. Strips of B.S bark are peeled away, yielding a gummy oleo-resin (9). B.S contains oils and β-boswellic acid, 3-O-acetyl-β-boswellic acid, 11-keto-β-boswellic acid and 3-O-acetyl-11-keto-β-boswellic acid (10). B.S has a variety of pharmacological effects, particularly anti-hyperglycemia effect (11), antioxidant effects, may have beneficial effects in peptic ulcer (12) and also anti-inflammatory effects in asthma (13), inflammatory bowel disease (14), cancer (15), and osteoarthritis (16).The hexane and alcoholic extracts of B.S protect liver against toxicity and damage induced by carbon tetrachloride, paracetamol or thioacetamide (17). Preparations that contain B.S oleo gum resin reduce blood sugar in STZ-induced diabetic rats (11). In another study, the root and leaf extracts of Boswellia glabra also reduced blood glucose levels in diabetic rats with alloxan (18). These effects of B.S made this plant a good candidate for studies on diabetes and its complications in the liver and the kidney.

## 2. Objectives

This study focused on the effects of *B.S* extract on diabetes complications in the liver and kidney and examined its impact on reproduction in diabetic animals.

## 3. Materials and Methods

B.S dried resin were purchased from herbal shop in 2011 and it was confirmed by pharmacognosy department school of pharmacy. Streptozotocin (STZ) from Sigma-aldrich corporation of America and TPTZ, Fecl_3_.6H_2_O, FeSO_4_.7H_2_O, HCL, Acetic acid, CH_3_COONa.3H_2_O and methanol were purchased from Merck.

All other reagents were of analytical grade.

### 3.1. Preparation of Plant Extract

Dried B.S oleo gum resin was added to hot distilled water on the magnetic heater for 1 hr, and then the extract was filtered. Solvent was removed using freeze dryer (operon). Resulting porous powder was stored in a refrigerator at 4-8°C till use.

### 3.2. Ferric-Reducing Antioxidant Power (FRAP) Assay

The antioxidant capacity of plant extracts was performed according to the method of Namjooyan et al. (19). The FRAP reagent included 10 mM TPTZ solution in 40 mM HCl, 20 mM FeCl_3_ solution and 0.3 M acetate buffer (pH 3.6) in proportions of 1:1:10(v/v). Then, 50 μL of each diluted extracts were mixed with 3 mL of freshly prepared FRAP reagent and the reaction mixtures were incubated at 37 °C for 4 min.

Absorbance at 593 nm was determined against distilled water blank. Aqueous solutions of ferrous sulfate (100–2000 μM) were used for calibration. Triplicate measurements were taken and the FRAP values were expressed as mmol of Fe (II)/g dry weight of plant powder (20).

### 3.3. Animals

Adult female Sprague-Dawley rats between 70-90 days of age with body weight ranging between 200-240 gm were used for the study. Animals were taken from experimental animal house of Ahvaz university of Medical Sciences. Animals were maintained under standard condition (temperature 20 ± 2, humidity 60 to 65 percent and in the 12-hour dark, lighting conditions) (7). Animals were fed with standard pellet diet and had free access to water. All the ethical issues were considered based on the Ahvaz Medical University Ethical Protocols (AMUEP) on animal experiments.

### 3.4. Induction of Experimental Diabetes

The animals were kept hungry for 8 hours before the injection of STZ. STZ was dissolved in sodium citrate buffer (0.09 M and pH 4.8). Diabetes induced by a single intraperitoneal administration of freshly dose of STZ (45 mg/kg body weight). One week after STZ injection, animals with blood glucose level above 250 mg/dl were taken as diabetic for the study. The blood sample was drawn from the lateral tail vein of rats and measured by portable glucometer (bionime model).

### 3.5. The Establishment of Pregnancy in Female Rats

For mating, the female diabetic and healthy male rats were placed together in the cage. Successful mating in rats is verified by the presence of a vaginal plug (VP) by microscope. Female rats were caged with syngeneic male rats and checked daily for the presence of vaginal plaque. Plaque positive female rat were selected and examined for the presence of sperm in vaginal smears. Only when both criteria of vaginal plaque and sperm presence were met, rats were considered to be at zero day of pregnancy (6, 21).

### 3.6. Experimental Procedure

VP positive rats were randomized into five groups of five rats each. Rats received vehicle or extract orally by gavage from the first pregnancy day until the seventeenth day as follows:

Group I: Healthy untreated rats

Group II: Diabetic control rats

Group III, IV and V: Diabetic rats given the doses of 200, 400 and 600 mg/kg of B.S extract, respectively.

Blood glucose was measured three times: including 7th days after STZ injection, after single dose administration of extract at the first day and at 17th day of study. Blood glucose measured half an hour after the administration of the extract or vehicle. In the 17th day of pregnancy, animals were scarified under anesthesia with ether, blood sample obtained from heart and collected in heparinated tubes to determine blood glucose level and HbA1C factor. The liver, kidney and uterus were quickly removed and washed with ice-cold isotonic saline. The liver and kidneys were placed in 10% formalin and the paraffin blocks of tissue prepared then cut in sections (3-5 μm thick). In order to evaluate possible morphological changes of liver and kidney, tissues of the different groups stained with hematoxylin and eosin (H and E). In the uterus the number of cicatricial plaques showing sites of previous implantation fetus were counted and the crown-rump length was measured (22).

### 3.7. Statistical Analysis

All the results were expressed as Mean± S.E for five animals in each group. Data were analyzed using analysis of variance (ANOVA) and post hoc test of Tukey’s. Differences below the 0.05 level were considered as statistically significant.

## 4. Results

**Figure 1:**
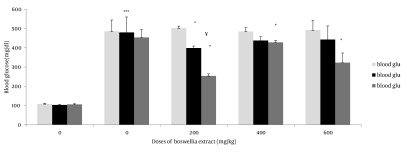
B.S aqueous extract effects on blood glucose in different days * Significant difference between blood glucose of diabetic group treated by 600 mg/kg BS extract in 17th day with 1st and 0 day P ≤ 0.05 ¥ Significant difference between blood glucose of diabetic group treated by 200 mg/kg BS extract in 17th day with 0 day P ≤ 0.01 * Significant difference between blood glucose of diabetic group treated by 200 mg/kg BS extract in 17 day with 1 day P ≤ 0.05 * Significant difference between blood glucose of diabetic group treated by 200 mg/kg BS extract in 1 day with 0 day P ≤ 0.05 * Significant difference between blood glucose of diabetic group treated by 400 mg/kg BS extract in 17 day with 1 and 0 day of diabetic P ≤ 0.05 *** Significant difference between HbA1c of diabetic group with that of control group P ≤ 0.001

**Figure 2:**
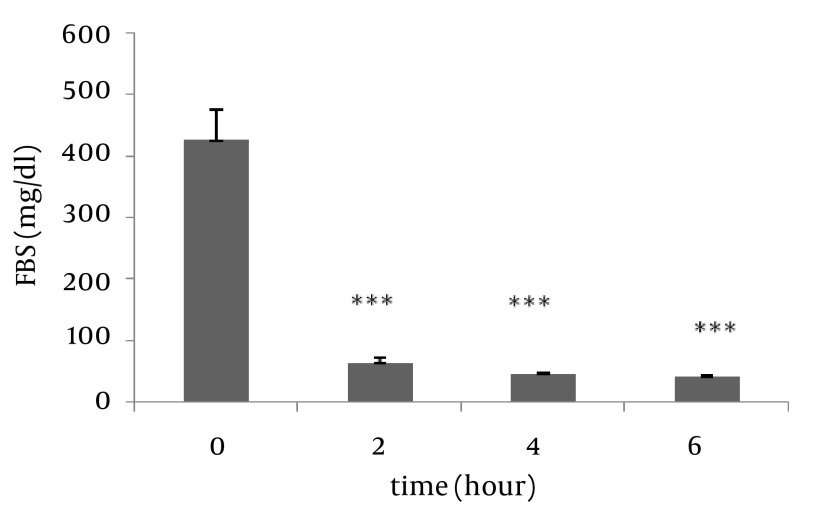
Glucose lowering effects of B.S extract (200 mg/kg) at different times. *** Significant difference between blood glucose of diabetic group treated by 200 mg/kg BS extract before and after 2, 4 and 6 hours. P ≤ 0.001

Antioxidant effect of B.S oleo gum resin extract were evaluated and compared with the standard control group. The FRAP value for B.S aqueous extract was 0.99 mmol Fe^2+^/ L. The mean blood glucose values in all groups are shown in Figure 1. As presented in Figure 1, diabetic control rats showed a significant increase in blood glucose and HbA1c compared to control group (P ≤ 0.001) (Table 1). Administration of B.S in diabetic rats significantly decreased the level of blood glucose and HbA1c at day 17 of gestation (P ≤ 0.01). Among the examined doses, the most effective dose of B.S extract was 200 mg/kg. In order to evaluate the time-course activity of 200 mg/kg dose, fasting blood glucose was measured at the 2, 4, and 6 hours after extract administration (Figure 2).

**Table 1:** HbA1c level in different groups

	HbA1c
Group I	3.58 ± .302
Group II	9.75 ± .3
Group III	5.6 ± 0.05
Group IV	7.7 ± 0.002
Group V	7.75 ± 0.57

**Table 2:** Distribution of variables in the mother and fetus experiences extracts of B.S

	Group I	Group II	Group III	Group IV	Group V
The number of VP at the day 0	5	5	5	5	5
The number of pregnant rats	3	5	1	-	1
The total number of embryos	21	47	12	-	9
The average number of fetuses per pregnant rat	7	9.4	12	-	9
The number of resorbed Embryos	0	9	0	-	0
Resorbed/ total embryos Ratio	0	0.19 a	0	-	0
The crown-rump length of embryos, cm	1.9 ± 0.09	1.54 ± 0.125	0.5 ±.0001 b		1.5 ± 0.0001
the crown-rump length of embryos, cm/ The total number of embryos Ratio	0.27 ± 0.03	0.23 ± 0.08	0.04 ± 0.001	-	0.16 ± 0.025

Comparison resorbtion/total embryos Ratio in the different groups:

^a^Significant difference between diabetic group with control group and diabetic groups receiving 200 and 600 mg/ kg B.S extract. P ≤ 0.001

^b^Significant difference between the crown-rump length of embryos in diabetic group receiving 200mg/ kg B.S extract with control group and diabetic groups receiving 600 mg/kg B.S extract. P ≤ 0.01

### 4.1. Histomorphologic Changes in Liver

**Figure 3:**
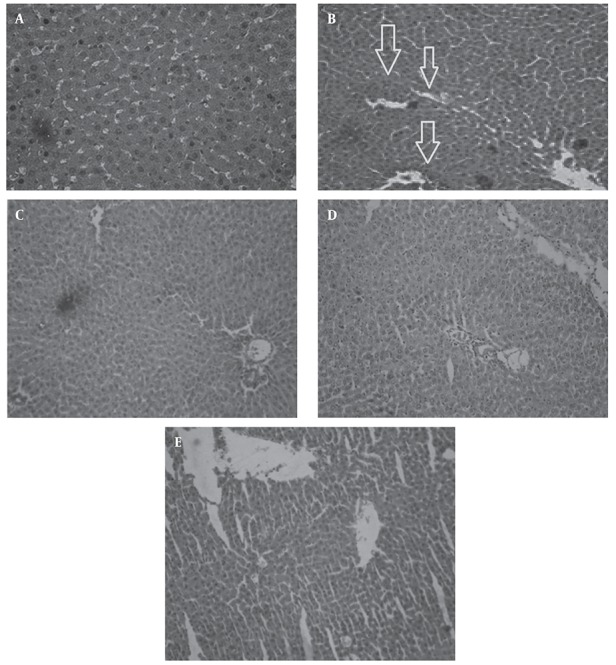
Histomorphologic changes in liver (tissues are stained with hematoxylin and eosin). A: Photomicrograph of liver from healthy animals receiving vehicle. (X = 100). B: Photomicrograph of liver from diabetic animals receiving vehicle. (X = 100). C: Photomicrograph of liver from diabetic rats treated by 200 mg/kg BS extract dosage (X = 100) D: Photomicrograph of liver from diabetic rats treated by 400 mg/kg BS extract dosage (X = 100) E: Photomicrograph of liver from diabetic rats treated by 600 mg/kg BS extract) dosage (X = 100)

The area around the port, hepatocytes, sinusoids, space around sinusoids, interstitial area, and areas around the central veins were observed in liver sections. In each slide ten fields were examined. In control group, endothelial cells around central vein are appropriately regular and normal and hepatocytes are normal, although some are staining more vulnerable but the core of some hepatocytes such as vesicular cytoplasm is eosinophilic and cells are seen with two nuclei (Figure 3). In diabetic group, size of hepatocytes was small, in addition to endothelial cells around central vein, cells with multi nucleus are gathered around central vein that have normal staining. The classic lobules around central vein in the cumulative core abnormality that occurs in the nucleus, which their origins are not clear whether endothelial cells or hepatocytes. Some parts of the sinusoids are swollen. Sinusoid spaces are quite inflated and dilated which could be due to decreased glycogen stores, it is not observed in all the tissues, but in some parts of this expansion is well evident. Fat accumulation is seen in some cells that can result in diabetes (Figure 3). In treated group by 200 mg/kg B.S extract: Hepatocyte and central vein are normal, the inflammation is not in sinusoid and nuclear accumulation is not seen around central vein, hepatocytes are small (Figure 3). In treated group by 400 mg/kg B.S extract: Cells are less eosinophilic, glycogen stores are less, central vein and cells are normal. There is not seen any inflammation, nuclear accumulation is not seen around central vein, but inflation is seen around some of them (Figure 3). In treated group by 600 mg/kg B.S extract: Inflammation is present in sinusoid cells around central vein and sinusoids are seen fully dilate (Figure 3).

### 4.2. Histomorphologic Changes in Kidney

**Figure 4:**
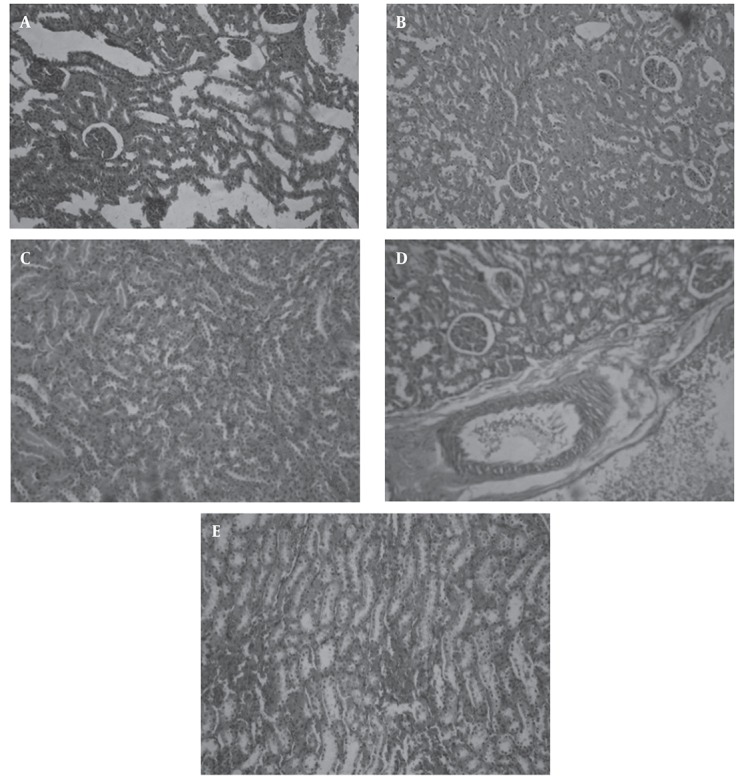
Histomorphologic changes in kidney (tissues are stained with hematoxylin and eosin). A: Photomicrograph of renal from healthy animals receiving vehicle (X = 100). B: Photomicrograph of renal from diabetic animals receiving vehicle (X = 100). C: Photomicrograph of renal from diabetic rats treated by of 200 mg/kg BS extract dosage (X = 100) D: Photomicrograph of renal of diabetic animals treated by 400 mg/kg BS extract dosage (X = 100) E: Photomicrograph of renal of diabetic animals treated by 600 mg/kg BS extract dosage (X = 100)

Distal and proximal tubules, Bowman capsule, Urinary tubules, glomeruli Bulb and malpighi body were examined in kidney. In control group, Cortexes and Bowman capsule are healthy, collecting tubes in the medullar appear normal (Figure 4). In diabetic group, Cell lyses occurred in the tubules of the kidney and cortex has been damaged, the distal and proximal tubules were destructed. The cortex of is not normal intact but Bowman’s capsule is not injured. Urinary space dilated, and the Malpighi body is wrinkled in some parts (Figure 4). In treated group by 200 mg/kg B.S extract: Cortex, Bowman capsule and collecting tubes in the medullar are normal (Figure 4c).In treated group by 400 mg/kg B.S extract, some glomeruli are damaged and wrinkled. Dilation can be seen in the space between the tubes and in the urine lumen in the cortex and medulla (Figure 4d). In treated group by 600 mg/kg B.S extract: Slightly dilatation can be seen in medulla, cortical veins are dilated, glomeruli shrinkage affected cortex (Figure 4e).

### 4.3. The Fetus Data

The pregnancy rate in group I to V was 60, 100, 20, 0, and 20% respectively. B.S probably could decreases pregnancy rate. Based on the results of diabetic rats received extract were not sufficient for argument the group I and II were used for discussion of fetus data. The average number of fetuses per pregnant rat in groups I and II was 7 and 9.4 respectively. The mean crown-rump length of fetus in groups I and II was 1.9 and 1.54 cm respectively. The fetus resorption in groups I and II was 0 and 0.19 respectively.

## 5. Discussion

Oxidative stress recently as one of the mechanisms in diabetes mellitus, that carbohydrate metabolism, lipids and proteins are affected, has been proposed.diabetes is characterized by increase oxidative stress and hyperglycemia was shown to directly induce oxidative stress by depleting natural anti-oxidants and facilitating the production of reactive oxygen species (ROS) under diabetic conditions ROS are produced through glycation reaction, which occurs in various tissues (25). Several changes and features are oxidative nature in people with diabetes or that are associated with the increased oxidative stress (26). Glycation of some compounds (27), and false –hypoxia induced by hyperglycemia (28), can be a position in the oxide and Reducing imbalances within cells to cause liver and kidney tissues.

It has been reported that the anti-oxidants vitamin C or vitamin E, protect beta cells from destruction in diabetic animal models (25). The present study shows that B.S extract could reduce blood sugar and HbA1c level in diabetic rats. B.S extracts at the dose of 200 mg/kg showed the highest effect compared with other doses. Our study shows that there reduction two hours after administration of the extract reaches its highest and up to six hours after administration of the extract is continuing.

Chronic high blood glucose increases free radicals production and oxidative stress which causes damage in other organs including the liver and kidneys. One of the most damaging effects of free radical initiated lipid peroxidation, is destruction of cell membranes. Histopathological changes in the liver caused by diabetes are including inflammation around the portal vein, fibrosis and vein enlargement, the hyperama in sinusoid, granular destruction, cell necrosis, the formation of macrovascular and microvascular vacuoles (2). In the histopathological study of liver in diabetic group, lymphocytic inflammation in the port areas, irregularities, apoptosis of liver cells, and dilatation of the sinuosoids were observed (Figure 3).

Glomerular hypertrophy occurs in diabetic nephropathy, hyper filtration occurs due to the enlargement of the glomeruli. The dimensions and size of renal glomeruli, glomerular area, mesenchymal matrix cells and glomerular basement membrane increases in diabetes, and a mild decrease occurs in size parenchymal matrix (1). Microscopic slides of the liver and kidney tissues demonstrates that B.S with has the effects anti-oxidant and anti-hyperglycemia Protective effects against damage caused by diabetes. B.S extract in doses of 200, and 400 mg/kg can largely reduce the harmful effects of diabetes, that including lymphocytic inflammation in the port areas, irregularities, apoptosis of liver cells, and dilatation of the sinuosoids were not observed. B.S extract at the dose of 600 mg/ml did not show a protective effect on liver and also cause cell death, inflammation and vasodilatation in sinuosoids space (Figure 3).

In the present study in groups treated by 200 and 400 mg/kg extract any inflammation and accumulation in the liver tissue was observed. All of symptoms that mentioned were improved in B.S treatment groups. Administration of 600 mg/kg of extract in the diabetic group had increased symptoms. The use of B.S extract in 200,400 doses could help prevent complications of diabetes in the kidneys (Figures 4 c and d). These complications were including: cell lyses occurred in the tubules of the kidney and cortex has been damaged, the distal and proximal tubules were destructed. The cortex of is not normal intact but Bowman’s capsule is not injured. Urinary space dilated, and the Malpighi body is wrinkled in some parts. (Figures 1 and 2 b)

B.S extracts used in appropriate doses, while having anti-hyperglycemia effect on blood sugar can be prevented complications of diabetes in liver and kidney. Boswellia serrata could probably decrease the pregnancy rate. This data suggests that diabetes increases the number of fetus, significantly fetus resorption, and decreases the fetus length. The crown-to-rump length is likely a reflection of a general environmental stress factor during embryonic growth (23). In previous studies conducted in this field has also been shown that fetuses of diabetic mothers give birth to smaller ones. In the diabetic groups a decrease was seen in the crown-rump length of embryos (24). Studies show that the rate of spontaneous abortion, miscarriage and perinatal mortality in diabetic mothers was higher in the poor glycemic control groups (5).
